# The association of upper limb sensorimotor capacity, everyday inpatient behavior, and the effects of neurorehabilitation in persons with multiple sclerosis and stroke: a mixed-design study

**DOI:** 10.1186/s12984-025-01586-z

**Published:** 2025-03-05

**Authors:** Philipp Gulde, Heike Vojta, Stephanie Schmidle, Peter Rieckmann, Joachim Hermsdörfer

**Affiliations:** 1 Chair of Human Movement Science, Department Health and Sport Sciences, TUM School of Medicine and Health, Technical University of Munich, Munich, Germany; 2 Center for Clinical Neuroplasticity, Medical Park SE, Bischofswiesen, Germany; 3 Friedrich-Alexander University, Erlangen-Nurnberg, Germany

**Keywords:** Stroke, Multiple sclerosis, Wearables, Activities of daily living, Neurorehabilitation

## Abstract

**Background:**

Quantifying and monitoring the sensorimotor state of persons with neurological disease by means of wearables in everyday life has been shown to be a promising approach. To date, the impact of physical activity volumes in fixed epoch approaches has been limiting the feasibility of kinematic analyses of everyday life upper limb use.

**Methods:**

Using acceleration and angular velocity signals from wrist-worn sensors, we collected data of healthy controls (n = 12) as well as persons with multiple sclerosis (n = 17) or stroke (n = 14) during everyday life during inpatient neurorehabilitation. An activity recognition algorithm was used to avoid physical activity volume dependencies that come with epoch-based approaches. Behavioral kinematics were compared between samples and associated with clinical test performance. Further, changes of sensorimotor capacity and behavioral kinematics during neurorehabilitation (n = 15) were analyzed.

**Results:**

Physical activity volume independence was achieved. Persons with neurological disease showed less activities and longer activity durations. Further, a PCA suggested three underlying components, namely: behavior, neurological state, and physical state. Components scores were lower (worse) for persons with neurological disease, except for behavior. However, component scores of persons with neurological disease showed great variability in all dimensions. Changes in sensorimotor capacity were partially associated with changes in behavioral kinematics, but effects of neurorehabilitation were mostly seen in outcomes associated with the physical state component.

**Conclusions:**

Persons with neurological disease showed neurological impairments as well as declines in the physical condition, which can to some extent be seen in behavioral kinematics. Neurorehabilitation appeared to rather affect the physical than the neurological state. By the novel approach using an activity recognizer instead of fixed epochs, it was possible show traces of sensorimotor capacity, as assessed by clinical tests, in kinematics of everyday life behavior.

## Introduction

The sensorimotor capacity of individuals with neurological disease, for instance stroke (cerebrovascular accident—CVA) or multiple sclerosis (MS), shows varying degrees of association with radiologic or electrophysiological diagnostic outcomes [6, 9, 14, 28]. Such metrics are not only costly but also cumbersome to gather and do not necessarily provide direct conclusions about capacity (*what people could do*; can apply to sensorimotor capacity in abstract tasks as well as to activities) and behavior (*what people do*) [28, 61]. For an accurate assessment of sensorimotor capacity, used to estimate the capability to carry out activities, direct measurements remain indispensable. Assessing various dimensions of sensorimotor capacity, such as strength, motor speed, and speed of information processing, yields reliable data with minimal effort [18, 24]. Although these methods have demonstrated clinical validity [16, 28], their external validity has not yet been fully established [41, 43, 57, 60].

Until now, many studies have utilized wearable sensors to quantify everyday life behavior (in different settings like home or inpatient) in persons with neurological disease [2, 4, 17, 25, 29]; [30, 31, 50, 57, 60, 62]. Some of these studies have explored the extent to which sensorimotor capacity, as assessed in the laboratory, translates into everyday life behavior [2, 17, 29, 30, 41, 57, 62]. However, the findings from these studies are limited. For instance, some studies highlight associations with the general condition of an individual, such as a strong association between impaired upper limb activity and gait capacity [2], while other studies demonstrate that variations in upper limb sensorimotor capacity correspond to similar lateralities in behavior (i.e., hand use in an inpatient setting) [30, 43, 62]. Additionally, some research groups have examined the association between everyday life upper limb use and rehabilitation outcomes [39, 40, 60], which is crucial as neurorehabilitation aims to enhance everyday life functioning, rather than just improving clinical test performance. As a limitation, these studies were primarily conducted in stroke populations. Meaningful gains in function within short time windows (e.g., weeks) are not reliably found in (chronic) stroke [12, 39, 43, 60]. Therefore, investigations of potential translations from gains by rehabilitation measures into everyday life behavior (*performance*) are limited. Another noteworthy aspect is that commonly the physical activity volume is assessed, yet it can be significantly influenced by confounding factors such as psychological state, age, or learned non-use [25, 30] in addition to facilitators and barriers in the environment [61]. Further, it has to be taken into account that (first) stroke comes in a sudden and sensorimotor capacity additional suffers from detraining (non-/less-use) [11, 47, 53] that could, additionally, mask concurrent gains by neurological recovery. In contrast, persons with MS suffer from a progressive disease that reacts to training (use) [28] and therefore, remaining capacity could develop (or deteriorate) more heterogenous than the disease would dictate. Some evidence on different patterns of impairment is given in quality of life [45] and mobility and postural control [10, 58] research. To gather knowledge on the sensorimotor capacity of a limb by an observation of its everyday use, not only the frequency of use, but also its quality should be assessed, as quality refers to the coordinative abilities that allow movements after all and can be strongly associated with physical activity levels but can be anticipated less prone to confounders like motivation [30]. Previous studies have demonstrated that kinematic analyses of upper limb movements in complex activities of daily living (ADL) provide a valid and sensitive method to gather information on the functional status in persons with cognitive and/or bodily impairments [19, 54]. Kinematic analyses offer to not only quantify the volume of behavior, but also to quantify other aspects, i.e., the intensity and coordinative qualities, of behavior [19, 59]. Therefore, it is imperative to explore the potential of kinematic analyses of upper limb movement signals in everyday life settings. In a previous study, kinematic use-lateralities (behavior; actual use), assessed by wearables during everyday life in an inpatient setting, were well-associated with lateralities in lab-based sensorimotor tests like finger tapping or grip strength (capacity; potential use) in persons with stroke [30]. However, in a model considering the three dimensions of *volume*, *intensity*, and *qualit*y, the observed impairment in the sense of pronounced lateralities was found to be one-dimensional; meaning that persons with impairments showed stronger lateralities in the sensorimotor capacity tests in lab, as well as considering all three dimensions of (inpatient) everyday life use. This indicates that using fixed epochs for kinematic analyses could be strongly influenced by the use volume. Another study conducted by this workgroup analyzed (inpatient) everyday life movement kinematics using wrist-worn sensors in persons with MS but reported rather weak associations with sensorimotor capacities [29]; however, persons with MS revealed more specific associations between behavior kinematics and sensorimotor capacity that indicate that the link of sensorimotor capacity with everyday life could be different between MS and stroke. Still the association between sensorimotor capacity and behavior kinematics appear to be rather weak. We argue that using fixed time intervals for kinematic analysis (e.g., 60s epochs) may fail to account for the likelihood that everyday life may not predominantly consist of a set of prolonged activities (i.e., ≥ 60s) [17, 25, 29, 59]. For instance, when analyzing a 7s activity, such as drinking from a glass [1], within a 60s epoch, almost 90% of the time-series data may not correspond to the activity of drinking from a glass. In contrast, activities like walking outside can easily fill such epochs. This discrepancy can result in analyses being skewed by the overall volume of activity recorded rather than the specific activity of interest. Therefore, our novel approach was to identify the onsets and offsets of movements and kinematically quantify the signal, independent of its length. Therefore, longer bouts of cyclic activities, such as engaging in sports or the arm swing when walking long distances, as well as times of inactivity would not bias the gathered distribution of collected activities, as they are weighted equally alongside both short and long activities and exclude inactive intervals. However, especially persons with impaired sensorimotor capacity often exhibit movement fragmentation, such as short breaks or hesitation, during the execution of an ADL [19]. Therefore, it is not avoidable to set an arbitrary threshold that classifies inactivity as an activity’s offset or a prolonged hesitation within an activity. Further, the minimum duration that leads to a positive activity classification needs to be set in order to exclude external disruptions and twitches.

The lower extremities enable humans to move their body mass towards objects, while the upper limbs perform the interactions, such as preparing food, opening doors, or washing hands. Thus, the interaction between the hands and the environment or the body has to be considered a key factor. A common approach for assessing this interaction involves detecting functional postures, i.e., the horizontal positioning of the forearm [15, 17, 41]. Although being a powerful approach, interactions with the own body that can be scratching oneself, dressing, or toying with objects in the palm of one’s hand would not be captured. All those movements, whether short or long, planned or spontaneous, arise from a cost function (comprising of costs/investment, success rate, value/gain, and costs of failure) that is based on the sensorimotor capacity, similar to the “threshold hypothesis” of Schwerz de Lucena et al. [57]. Therefore, especially small, rather spontaneous movements could give information on the functional status of a limb. To capture those, we propose an algorithm that expects wrist rotation (to exclude gait) and translation (to exclude tremor) with a minimum duration of 400ms and a maximum allowable break or hesitation of 1s. The 400ms are based on the duration of pointing movements of young, healthy adults in Kornatz et al. [36]. Utilizing smartwatches, we captured the kinematics of (inpatient) everyday life behavior of persons with MS as well as persons with stroke during their inpatient stay at a neurorehabilitation facility. We compared their behavior kinematics with those of healthy adults, related the behavior kinematics to the participants’ sensorimotor capacity, and lastly examined whether the changes in sensorimotor capacity and behavior kinematics observed during rehabilitation were correlated. We used a mixed sample (MS & stroke), since it has been previously shown that rehabilitation has a significant, positive impact on the sensorimotor capacity of persons with MS [24, 34] and therefore, we would still be able to show feasibility, even if the persons with stroke should not positively react to neurorehabilitation. Through this new approach, we aim to enhance our understanding of the external validity of sensorimotor capacity tests, elucidate the specific and potentially disease-dependent effects of neurorehabilitation, and gain deeper insights into everyday upper limb use. As Dusfour et al. [17] and Schwerz de Lucena et al. [57] have already shown, humans use their hands thousands of times per day, e.g., approx. 10 functional uses per minute resulting in several thousand functional uses per day in Dusfour et al. [17], but not every use is connected to brushing one’s teeth, preparing a cup of coffee, or buttoning a shirt. Therefore, we argue that everyday life most likely comprises of a large amount of short, not directly ADL-associated movements, which could hold meaningful information on an individual’s functional status.

Summarizing, the purpose of this study was to kinematically analyze (inpatient) everyday behavior in different neurological diseases in order to examine the association of behavior and sensorimotor capacity, to further examine potentially disease-dependent effects of neurorehabilitation, and in addition to examine the underlying dimensions that are connected with everyday upper limb use. We hypothesized that the new approach allows to describe movement quality and intensity independent of the physical activity volume and that persons with neurological diseases show different behavior kinematics, especially less activities of prolonged duration (as an indicator of reduced task performance). Moreover, we hypothesized that sensorimotor capacity and behavior are associated and that the neurological samples differ in their sensorimotor capacity. We further hypothesized that both neurological samples react differently to neurorehabilitation, with persons with multiple sclerosis showing positive effects on sensorimotor capacity and persons with stroke showing no detectable changes within the short time window of neurorehabilitation. Last, we hypothesized that changes in sensorimotor capacity translate into (inpatient) everyday behavior.

## Methods

### Sample

Our convenience sample consisted of 12 healthy adults (CON), 17 persons with MS (MS), and 14 persons with stroke (CVA). Longitudinal data, i.e., data covering an interval of 10-27d of neurorehabilitation, were available of 8 persons with MS (EDSS 3.2 ± 1.2 with a range of 2.0–6.0) and 7 persons with stroke (4.9 ± 6.7 months since stroke with a range of 1–17 months). The limited sample sizes and the limited availability of longitudinal data is based on time constraints (the research facility at the clinic was closed due to economic reasons). Persons in the stroke sample were older than in the other samples. Both neurological samples exhibited similar degrees of sensorimotor impairments based on the Watzmann Severity Scale (WSS) (p = 0.796, Table 1) [24]. Details of the samples can be found in Table 1. Inclusion criteria were a diagnosed MS or a unilateral stroke in the subacute to chronic stage. Exclusion criteria were psychiatric disorders, including dementia, severe psychological disorders, drug abuse, orthopedic impairments of the upper limbs, and neurological comorbidities. All participants were recruited at an inpatient neurorehabilitation facility in Germany. All subjects gave written informed consent to participate in the study. Ethical approval was given by the ethical committee of the School of Medicine and Health of the Technical University of Munich (identifier: 478/19 S-SR).

**Table 1:** Sample characteristics with means, standard deviations, and ranges

Parameter	Healthy adults (CON) (n = 12)	Persons with MS (MS) (n = 17)	Persons with stroke (CVA) (n = 14)	Comparison	
Age [a]	46.5 ± 11.8 (29–63)	51.8 ± 10.9 (31–64)	61.0 ± 15.9 (42–83)	CON vs. MS CON vs. CVA MS vs. CVA	p = 0.129 p = 0.012, d = 1.23 p = 0.103
Sex	58% Female 42% Male	100% Female 0% Male	57% Female 43% Male	CON vs. MS CON vs. CVA MS vs. CVA	p = 0.089 p > 0.999 p = 0.125
WSS	1.9 ± 0.5 (1.0–2.9)	3.2 ± 1.1 (1.7–4.6)	3.4 ± 0.8 (2.5–4.9)	CON vs. MS CON vs. CVA MS vs. CVA	p = 0.007, d = 2.46 p = 0.002, d = 2.70 p = 0.796
Repeated measures interval [d]	Not applicable	20.0 ± 5.6 (11–27) n = 8	18.9 ± 6.4 (10–26) n = 7	MS vs. CVA	p = 0.721

WSS: The Watzmann Severity Scale is a sensorimotor estimate of the expanded disability scale score (EDSS) [24, 37]. Higher values indicate a more severe sensorimotor impairment. The repeated measures interval is the time spent in inpatient neurorehabilitation between the first and second assessment. Comparisons were made with independent t-tests and chi-squared tests. Effect-sizes are reported with Glass’ delta (d). a is the symbol for year

### Procedure and equipment

Healthy adults (CON) were assessed once. Persons with MS (MS) and stroke (CVA) were assessed once close to their inpatient rehabilitation entry and once close to their release.

#### Sensorimotor capacity

For sensorimotor capacity assessments, an isometric hand dynamometer (Deyard EH101, Deyard Corp.) was used to measure grip strength. The best of two trials of each hand was used and hand- and sex-specific z-scores were computed based on the performance of the control sample. To assess manual dexterity, participants executed the nine-hole peg test once with each hand. The inversed trial duration was computed as a performance measure (i.e., speed) to achieve a normal distribution, provide a parameter suitable for linear regression, and account for cases where task execution was not possible, resulting in a speed of 0/s [30]. For motor speed, the maximum upper limb tapping frequency of each arm was measured using a smartphone application, which recorded the tapping rate over 10s) [26]. Reaction times were gathered using the participant’s better functioning upper limb (free choice of participants) over 10 trials in response to a visual stimulus using a smartphone application [24]. All smartphone tests were conducted on a Samsung Galaxy A7 2018 (Samsung Electronics, KR) with a 6-inch screen.

#### Kinematic performance

Behavior kinematics were collected using Huawei 2 4G (Huawei Ltd., CN) smartwatches, worn on each wrist, for one day during inpatient neurorehabilitation. Data acquisition was realized by custom-made software, called 2MPAC (*Technical University of Munich Physical Activity Counter*), developed using Microsoft Visual Studio (2019; Microsoft Corp., US) for the Android operating system. The software captured data from the smartwatches’ three-axial accelerometer (100Hz), gyroscope (25Hz), and the integrated acceleration-based pedometer. Data were smoothed using a 140ms moving average [21].

### Activity recognition

**Fig. 1:**
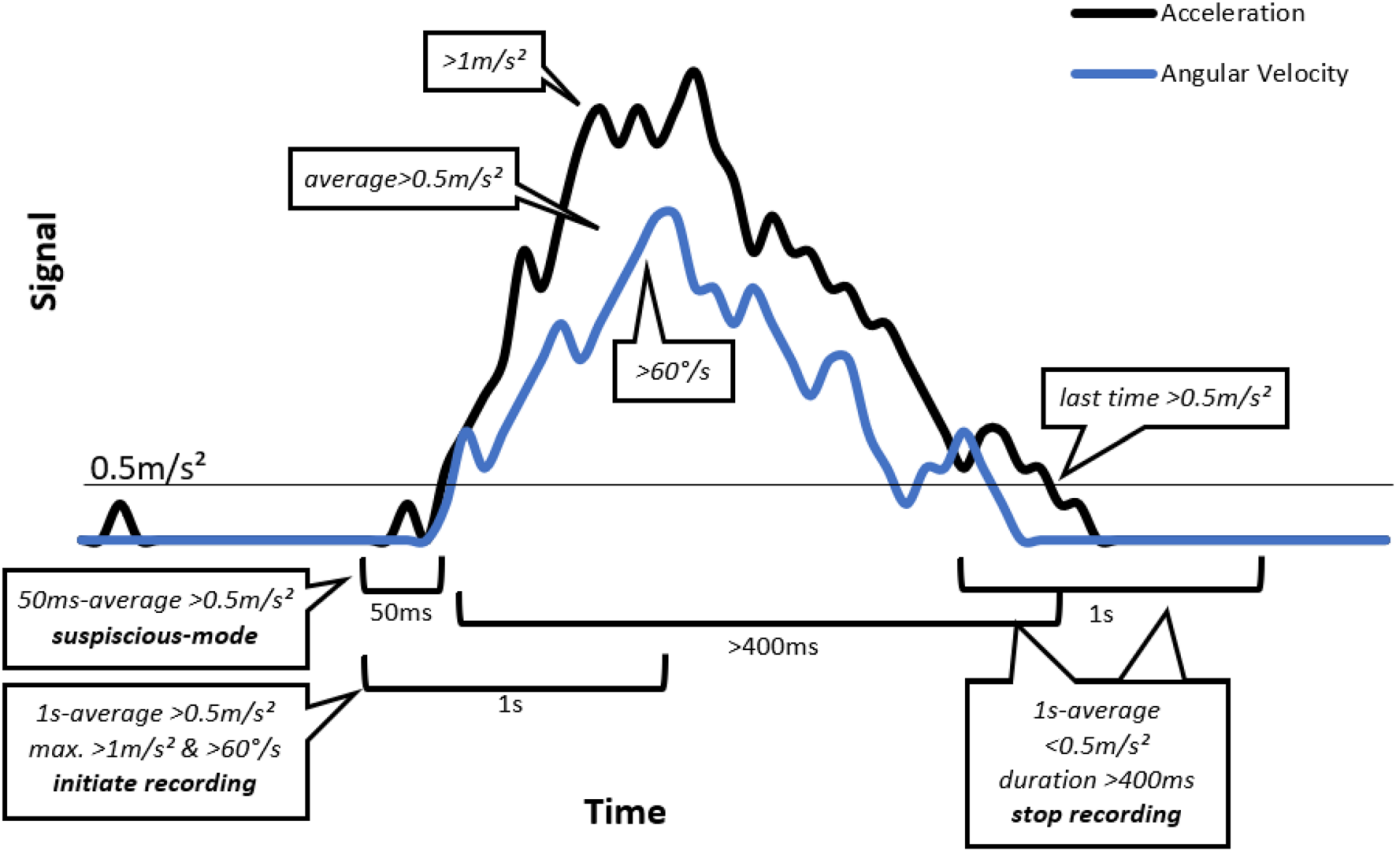
Algorithm for the detection of the start and end of activities. If, within the last 50 ms an average acceleration of 0.5 m/s^2^ is surpassed, a suspicious-mode is entered. In this mode, recording is initiated if an 1s-average of 0.5 m/s^2^ and maxima of 1.0 m/s^2^ and 60°/s are reached. The recording ends if the average of the last second is below 0.5 m/s^2^ and the duration from start to the last value of more than 0.5 m/s^2^ is exceeding 400 ms. The end of recording is set to the last value greater than 0.5 m/s^2^. If, within the suspicious-mode, thresholds are not met, no recording is initiated and the program returned to waiting

For activity recognition, three distinct modes were defined: *waiting, suspicious,* and *recording*. Initially, the smartwatch continuously stored the most recent 50ms of sensor data in a state referred to as *waiting*. If an acceleration threshold of an average of 0.5 m/s^2^ [29, 54] during this period was detected, 2MPAC transitioned into a *suspicious* mode, in which sensor data were temporarily stored for up to 10s [17]. If, within the last second, the maximum acceleration was higher than 1 m/s^2^ [54], the maximum angular velocity exceeded 60°/s [29], and an average acceleration of at least 0.5 m/s^2^ [29, 54] remained, 2MPAC initiated *recording*. If these conditions were not met, 2MPAC returned to *waiting*. During recording, 2MPAC continuously monitored whether the average acceleration within the last second fell below 0.5 m/s^2^ [29, 54]. In this case, and if the recording exceeded a duration of 400 ms [36], the time interval from the beginning to the last acceleration data point surpassing 0.5 m/s^2^ was automatically subjected to kinematic analysis. The results of this analysis were logged in a file, after which 2MPAC returned to *waiting*. Note that the maximum length of movement breaks or hesitations was limited to 1 s. The algorithm is displayed in Fig. 1.

### Parameters—Lab-based sensorimotor capacity tests

Note that metrics were derived for each of the hands/upper limbs.

**Peg test performance** [1/s]: The inversed trial duration of the nine-hole peg test execution as an estimate of manual dexterity. Higher values indicate better performance [30].

**Grip strength**: The hand- and sex-specific z-scores of the kilogram equivalent of the maximum grip strength using an isometric handheld dynamometer (best of two trials) as an estimate of muscular strength. Higher values indicate better performance [24].

**Tapping frequency** [Hz]: The tapping frequency over 10s as an estimate of motor speed. Higher values indicate better performance [26].

**Reaction times** [ms]: The average reaction time over 10 trials using a visual stimulus as an estimate of information processing speed [18, 24]. Higher values indicate worse performance.

**Watzmann Severity Scale** (WSS): A sensorimotor estimation of the expanded disability status scale [24, 37] based on grip strength, upper limb tapping, postural control, and gait quality. Higher values indicate a more severe sensorimotor impairment.

### Parameters—2MPAC metrics

Note that metrics were derived for each of the hands/upper limbs.

**Activities per minute** [1/min]: The number of recognized activities divided by the wear time of the smartwatch. Higher values indicate more interactions with oneself and the environment [57].

**Activity duration** [s]: The average duration of activities. Higher values indicate a slower execution of activities (e.g., by sensorimotor impairment) [19].

**Mean amplitude deviation** (MAD) [mg]: The average distance of acceleration data points to their mean in milli-g as an estimate of movement intensity. Higher values indicate more intense movements [55]. MAD in fixed-epoch approaches is commonly used to estimate the energy expenditure [7].

**Mean acceleration** [m/s^2^]: The average acceleration during an activity. Higer values indicate more intense movements.

**Maximum acceleration** [m/s^2^]: The maximum achieved acceleration within an activity. Higher values indicate more intense movements, excessive values indicate jerky movements, e.g., by impaired movement dampening of translational movements.

**Mean angular velocity** [°/s]: The average angular velocity during an activity. Higher values indicate more intense wrist involvement (manipulation).

**Max angular velocity** [°/s]: The maximum achieved angular velocity within an activity. Higher values indicate more intense wrist movements (manipulation), excessive values indicate jerky movements, e.g., by impaired movement dampening of rotational movements.

**DFT frequency (translation)** [Hz]: The frequency with the highest weight in the power spectrum derived from a discrete Fourier transformation of the acceleration data of an activity. Higher values indicate quicker translational movements (i.e., with the arm) within an activity.

**DFT weight (translation)**: The highest weight of the power spectrum derived from a discrete Fourier transformation of the acceleration data of an activity. Higher values indicate smoother (well-coordinated) translational movements within an activity [5, 22].

**DFT frequency (rotation)** [Hz]: The frequency with the highest weight in the power spectrum derived from a discrete Fourier transformation of the angular velocity data of an activity. Higher values indicate quicker rotational movements (i.e., with the hand) within an activity.

**DFT weight (rotation)**: The highest weight of the power spectrum derived from a discrete Fourier transformation of the angular velocity data of an activity. Higher values indicate smoother (well-coordinated) rotational movements within an activity [5, 22].

**Steps per minute** [1/min]: The total number of recognized steps by the in-built pedometer divided by the wear time as an indicator of physical activity volume. Higher values indicate higher physical activity volumes. This parameter was assessed to check independence of behavior kinematics from the physical activity levels of participants. This parameter is gathered independently from the 2MPAC activity recognition algorithm.

Note that for behavior kinematics, the average of all logged activities was used to represent the general behavior (except activities per minute and steps).

### Statistical analyses

#### Hypothesis I: Independence of behavior kinematics from physical activity volume

Independence of the approach from the physical activity volume was tested by a correlation of the mean amplitude deviation (MAD) with steps in healthy adults (CON). In fixed-epoch approaches, MAD is very strongly associated with the energy expenditure [7], therefore, the hypothesis would anticipate a close-to-zero correlation.

#### Hypothesis II: Differences of behavior kinematics between the samples

Independent, two-sided t-tests were computed on group-level to give an easy to grasp picture of the samples’ behavior kinematics. For upper limb use and component scores analyses (PCA) repeated measures analyses of variance (anova) with the within-subjects factors hand (dominant or non-dominant) and lateralized impairment (affected body side based on self-report with verification by reduced sensorimotor capacity) and the between-subjects factors group (i.e., sample) and biological sex (and all their respective interactions) were computed.

#### Hypothesis III: Association of lab-based sensorimotor capacity and behavior kinematics

Simple correlations were calculated to preliminary explore the relationship between sensorimotor capacity and behavioral kinematics, resulting in a correlation matrix. For correlational analyses, every limb was treated as an instance, same as every assessment point. To control for dependent datapoints, repeated measures correlations were used [3]. In order to better understand the association of sensorimotor capacity, person characteristics (e.g., age), and behavior kinematics, an explorative principal component analysis (PCA) was computed. We used a varimax rotation. The number of components was derived from a scree plot. The Kaiser–Meyer–Olkin criterion (KMO) was set to a minimum of 0.7, and minimum communalities to 0.5. To improve readability, latent components were already labeled in the results section. The labeling was based on the strongest factor loadings, but already incorporated interpretation of the results to some extent.

#### Hypothesis IV: Differences of sensorimotor capacity between the samples

Independent, two-sided t-tests were computed on group-level to give an easy to grasp picture of the samples’ sensorimotor capacity. For sensorimotor capacity and component scores analyses (PCA) repeated measures analyses of variance (anova) with the within-subjects factors hand (dominant or non-dominant) and lateralized impairment (affected body side based on self-report with verification by reduced sensorimotor capacity) and the between-subjects factors group (i.e., sample) and biological sex (and all their respective interactions) were computed. Note that lateralized impairment affects most subjects of the neurological samples and therefore, group rather represents the general physical state of a person (only neurological samples can report lateralized impairments), lateralized impairment the neurological state, and the interaction of group and lateralized impairment the disease-specific state.

#### Hypothesis V: Different reaction of neurological samples to neurorehabilitation

The general effect of neurorehabilitation was tested by dependent, two-sided t-tests.

#### Hypothesis VI: Translation of sensorimotor capacity changes to behavior kinematics

Correlations between relative changes (differences between measurement time points divided by the first time point) were computed for sensorimotor capacity and behavior kinematics to check for translations of sensorimotor capacity changes to everyday behavior.

#### Thresholds, effect-sizes, and software

α was set to 0.05. For tests serving the purpose of an overview (e.g., correlation matrix), we did not apply α-level corrections. Effect-sizes are reported as the coefficient of correlation (r) and as the coefficient of determination (R^2^) for correlations, as generalized eta^2^ (ges) for anovas, as Glass’ delta (d) for comparisons with CON and as Cohen’s d for comparisons between MS and CVA. All statistical tests were run in RStudio (R 4.0.5, RStudio 1.4.1106; RStudio PBC, US).

## Results

As a result of technical issues, we lost one recording (dominant hand) of a person with MS and one recording was limited to 131 min, resulting in a total of 85 recordings. The recordings had an average time span of 479 min ± 97 min (131–688 min).

### Independence of behavior kinematics from physical activity volume

**Fig. 2:**
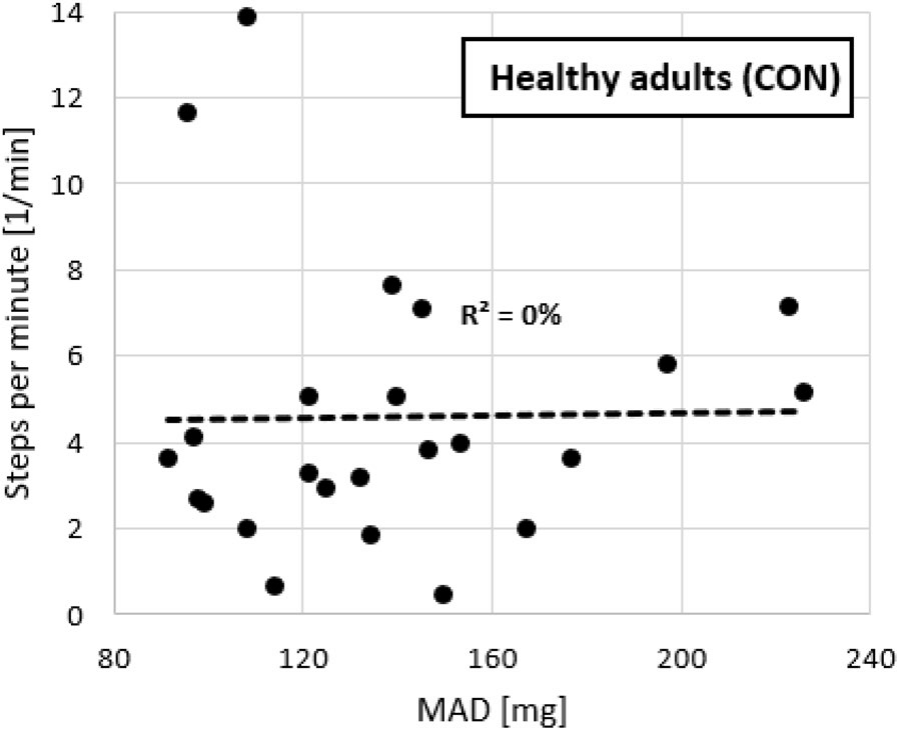
Null association of behavior kinematics (mean amplitude deviation) and physical activity volume (steps per minute) in the control sample

To verify the assumption of independence of behavior kinematics from the physical activity volume, we examined the association between the average MAD and the average steps per minute within the healthy sample. No significant association was observed between these two measures (p = 0.934) (Fig. 2) (Table 1).

### Differences of behavior kinematics between the samples

**Fig. 3:**
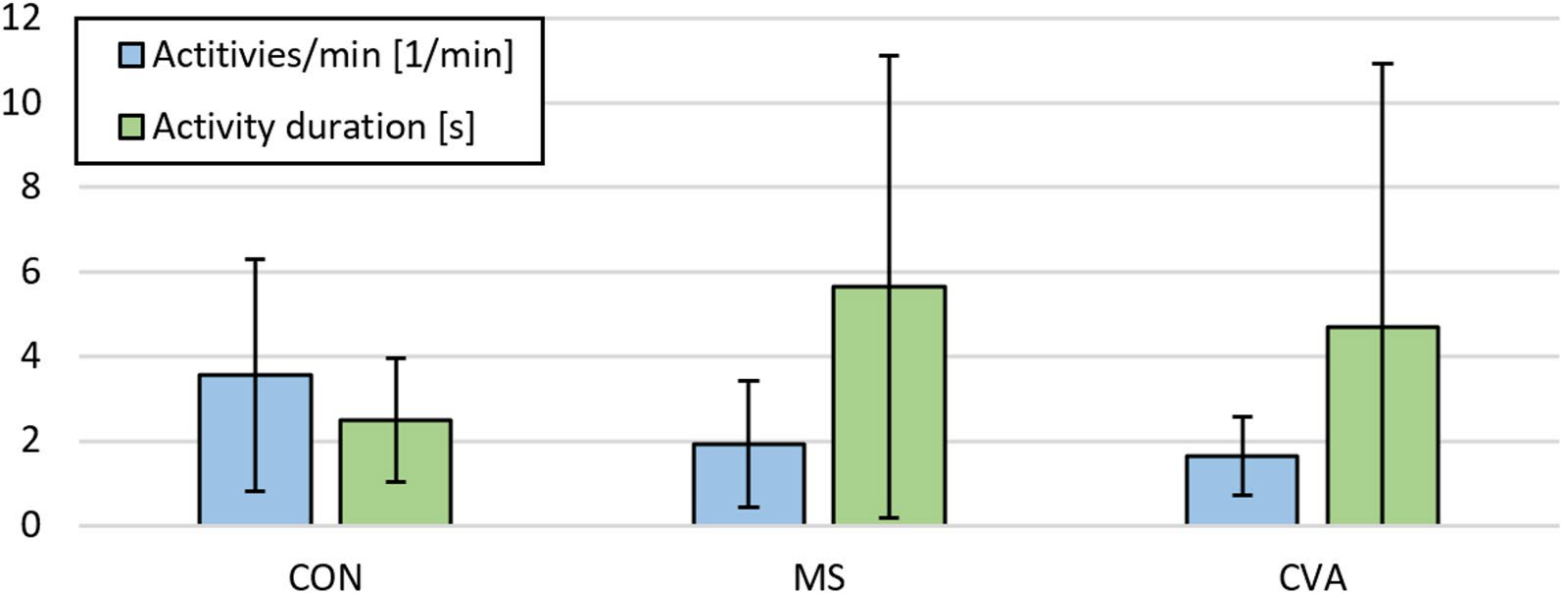
Means and standard deviations of activities per minute and activity duration in the samples. CON: healthy controls, MS: persons with multiple sclerosis, CVA: persons with stroke

**Fig. 4:**
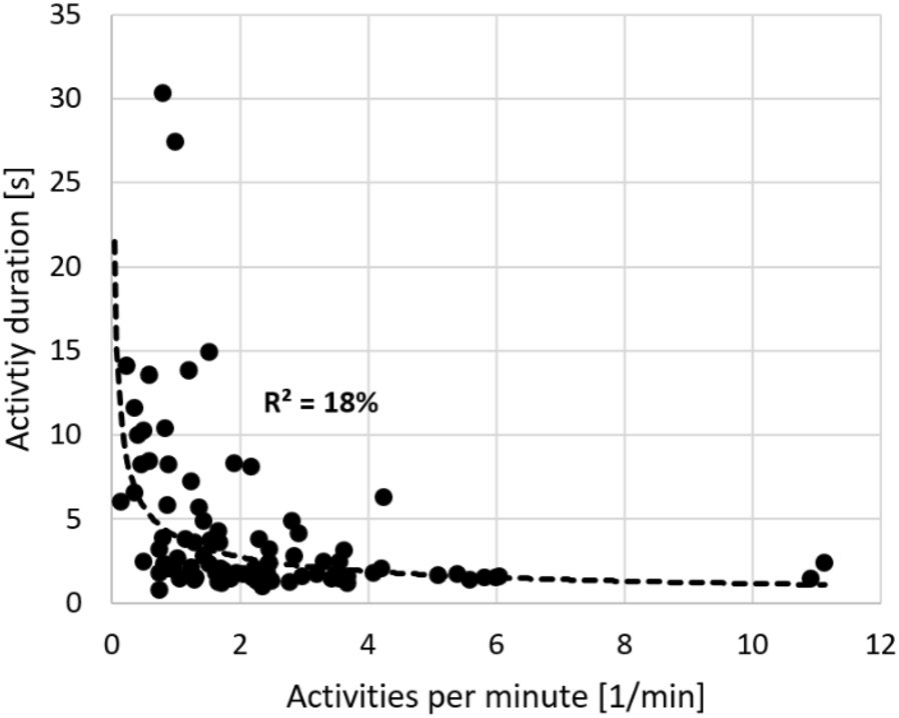
Association between activity duration and activities per minute across all participants (p < 0.001)

Healthy adults showed more activities per minute compared to persons with MS and with stroke. However, their activity durations were shorter than the durations observed in persons with MS (Table 2, Fig. 3). Across all participants, the number of activities per minute and activity durations were negatively associated (power function with r = − 0.43, p < 0.001) (Fig. 4). The time spent in activities per minute (activities per minute multiplied by activity duration) was similar across all samples (CON 7.7s/min ± 5.5s/min (12.8% ± 9.2%), MS 7.3s/min ± 6.0s/min (9.3% ± 10.4%), CVA 5.6s/min ± 6.3s/min (12.2% ± 10.0%); CON vs. MS p = 0.815; CON vs. CVA p = 0.203; MS vs. CVA p = 0.273). Table 2 gives an overview of the comparisons between the samples considering their behavior kinematics. Anovas revealed an effect of group on activities per minute (p < 0.001, ges = 0.20) and an effect of sex on MAD (p = 0.026, ges = 0.067; male > female). Further, anovas showed an effect of lateralized impairment on the maximum acceleration (p = 0.023, ges = 0.07; unimpaired > impaired), maximum angular velocity (p = 0.002, ges = 0.12; unimpaired > impaired), as well as DFT weight (rotation) (p = 0.018, ges = 0.08; impaired > unimpaired). A group effect was visible for the DFT frequency (translation) (p = 0.002, ges = 0.154; CON > MS & CVA) and an effect of lateralized impairment (p = 0.032, ges = 0.06; impaired > unimpaired) and sex (p = 0.016, ges = 0.08; male > female) for the DFT frequency (rotation).

**Table 2:** Behavior kinematics of the three samples

Parameter	CON	MS	CVA	Comparison	
Activities per minute [1/min]	3.56 ± 2.75	1.92 ± 1.49	1.63 ± 0.93	CON vs. MS CON vs. CVA MS vs. CVA	p = 0.012, d = 0.59 p = 0.003, d = 0.70 p = 0.362
Activity duration [s]	2.48 ± 1.48	5.65 ± 5.48	4.69 ± 6.24	CON vs. MS CON vs. CVA MS vs. CVA	p = 0.003, d = 2.15 p = 0.079 p = 0.532
MAD [mg]	138 ± 38	133 ± 36	114 ± 23	CON vs. MS CON vs. CVA MS vs. CVA	p = 0.649 p = 0.013, d = 0.61 p = 0.018, Cohen’s d = 0.61
Mean acceleration [m/s^2^]	0.93 ± 0.21	0.83 ± 0.09	0.81 ± 0.13	CON vs. MS CON vs. CVA MS vs. CVA	p = 0.046, d = 0.45 p = 0.019, d = 0.57 p = 0.378
Maximum acceleration [m/s^2^]	5.14 ± 1.29	5.63 ± 2.28	4.51 ± 1.25	CON vs. MS CON vs. CVA MS vs. CVA	p = 0.307 p = 0.080 p = 0.018, Cohen’s d = 0.60
Mean angular velocity [°/s]	111 ± 19	108 ± 22	94 ± 19	CON vs. MS CON vs. CVA MS vs. CVA	p = 0.582 p = 0.003, d = 0.88 p = 0.015, Cohen’s d = 0.64
Maximum angular velocity [°/s]	247 ± 37	273 ± 90	219 ± 58	CON vs. MS CON vs. CVA MS vs. CVA	p = 0.137 p = 0.044, d = 0.75 p = 0.007, Cohen’s d = 0.70
DFT frequency (translation) [Hz]	1.53 ± 0.07	1.47 ± 0.08	1.45 ± 0.11	CON vs. MS CON vs. CVA MS vs. CVA	p = 0.004, d = 0.86 p = 0.002, d = 1.12 p = 0.446
DFT weight (translation)	0.51 ± 0.05	0.46 ± 0.15	0.51 ± 0.12	CON vs. MS CON vs. CVA MS vs. CVA	p = 0.080 p = 0.994 p = 0.149
DFT frequency (rotation) [Hz]	1.17 ± 0.13	1.05 ± 0.30	1.14 ± 0.28	CON vs. MS CON vs. CVA MS vs. CVA	p = 0.047, d = 0.90 p = 0.661 p = 0.219
DFT weight (rotation)	0.58 ± 0.04	0.51 ± 0.12	0.55 ± 0.10	CON vs. MS CON vs. CVA MS vs. CVA	p = 0.008, d = 1.71 p = 0.232 p = 0.213

Means and standard deviations are given for the extracted kinematic parameters. Note that values in this table are not further differentiated by hand dominance and impairment

CON: Healthy adults, MS: persons with multiple sclerosis, CVA: persons with stroke. Comparisons were made with independent, two-sided t-tests. Effect-sizes are reported as Glass’ delta (comparison with CON) and Cohen’s d (comparison between neurological samples)

### Association of lab-based sensorimotor capacity and behavior kinematics

Table 3 illustrates the correlation coefficients between sensorimotor capacity tests and behavior kinematics. A better peg test performance was associated with more activities per minute and more intense translational and rotational movements. Grip strength was only associated with the intensity of translations. Tapping frequency was correlated with the intensity of translational and rotational movements. Higher reaction times were related to higher activity durations. None of the sensorimotor capacity parameters showed a significant association with DFT, i.e., coordination, parameters. Note that all associations were weak (|r|> 0.1) to moderate (|r|> 0.3).

**Table 3:** Coefficients of (repeated measures) correlation between sensorimotor capacity and behavior kinematics

	Peg test performance	Grip strength	Tapping frequency	Reaction times
Activities per minute	0.28*	0.20	0.15	0.13
Activity duration	0.04	0.03	0.01	0.23*
MAD	0.39*	0.22*	0.48*	− 0.15
Mean acceleration	0.32*	0.20	0.37*	− 0.20
Maximum acceleration	0.32*	0.21	0.40*	− 0.04
Mean angular velocity	0.33*	0.17	0.42*	− 0.03
Maximum angular velocity	0.34*	0.21	0.40*	0.09
DFT frequency translation	− 0.07	0.05	0.09	− 0.09
DFT weight translation	− 0.11	− 0.09	− 0.12	− 0.08
DFT frequency rotation	− 0.13	− 0.10	− 0.10	− 0.12
DFT weight rotation	− 0.15	− 0.07	− 0.12	− 0.11

Significant associations (p<0.05) are highlighted with an * asterisk

Note that higher values in peg test (inversed trial durations) indicate a better performance

MAD: mean amplitude deviation

The principal component analysis included the following variables (based on KMOs ≥ 0.70): age, a dummy variable for impaired body side (0 = less/unimpaired, 1 = (more) impaired), the WSS, the inversed peg test trial duration, grip strength, tapping frequency, activity duration, maximum acceleration, DFT weight of translation, maximum angular velocity, DFT frequency of rotation, and DFT weight of rotation. The model had a measure of sample adequacy of 0.82 (KMOs of 0.74–0.92), three components (eigenvalues 4.79, 2.62, 2.19), and 0.80 explained variance. Communalities were between 0.60 and 0.92. The factor loadings can be found in Table 4.

**Table 4:** Rotated component loadings of participant characteristics and the sensorimotor capacity and kinematic parameters

Parameter	Rotated component 1 “Behavior”	Rotated component 2 “Neurological state”	Rotated component 3 “Physical state”
Age	− 0.07	− 0.29	**− 0.71**
Grip strength	− 0.09	0.12	**0.88**
Peg test	− 0.11	**0.60**	**0.60**
WSS	− 0.02	**− 0.59**	**− 0.63**
Impaired body side	− 0.09	**− 0.82**	− 0.31
Tapping frequency	− 0.11	**0.90**	0.23
Activity duration	**− 0.89**	− 0.12	− 0.01
Maximum acceleration	**− 0.76**	0.41	0.07
Maximum angular velocity	**− 0.79**	0.38	0.08
DFT weight translation	**0.95**	− 0.03	0.00
DFT weight rotation	**0.96**	− 0.07	0.00
DFT frequency rotation	**0.96**	0.00	− 0.03

In all components, higher component scores indicate a better state/performance

Note that higher values in peg test (inversed trial durations) indicate better a performance. Loadings above 0.50 are marked in bold to indicate the main loadings of the components

**Fig. 5:**
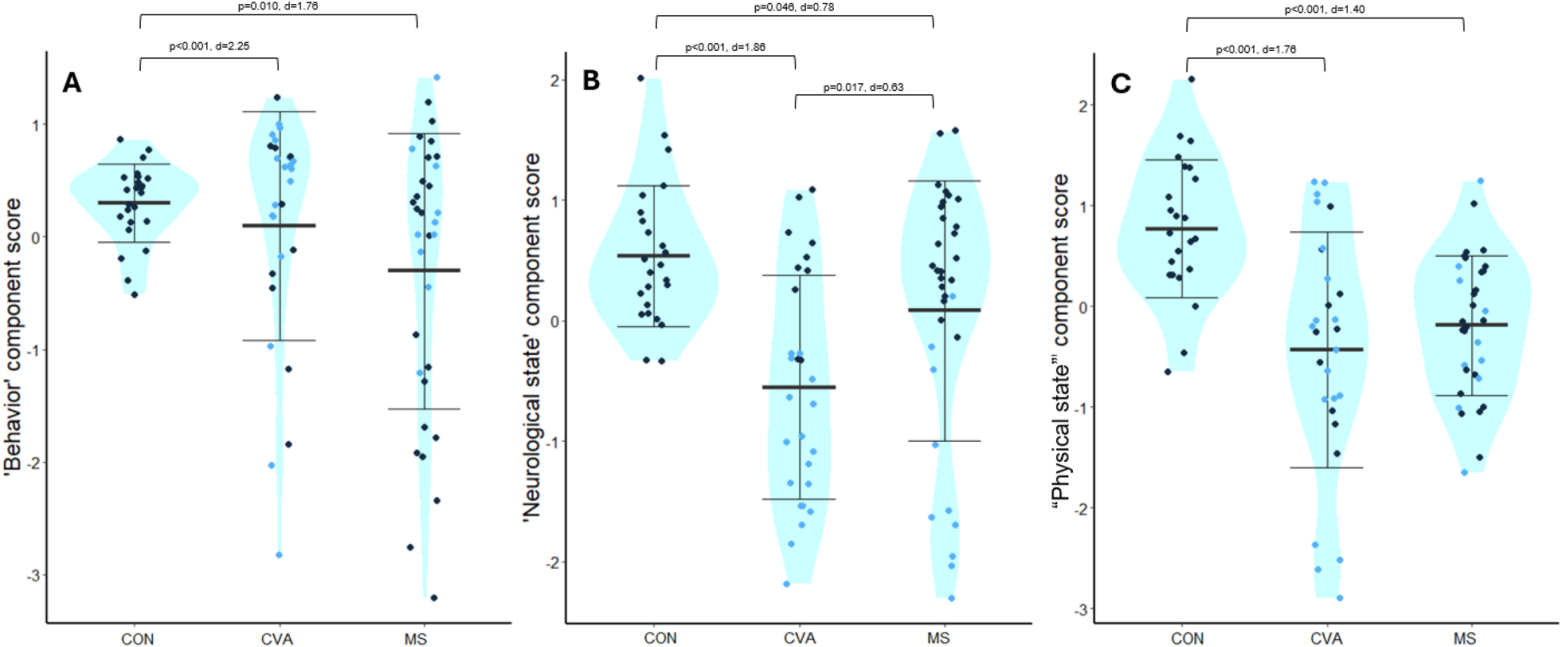
Distribution of individual component scores. Blue dot fillings indicate lateralized impairments. Bold bars indicate the mean, error bars 1 standard deviation. Comparisons on group levels are indicated with p-values and effects sizes (Glass’ delta for comparisons with CON, Cohen’s d for comparisons between MS and CVA). **A** “Behavior” component. Higher values indicate better performance. **B** “Neurological state” component. Higher values indicate a better status. **C** “Physical state” component. Higher values indicate a better status. CON: healthy controls, MS: persons with multiple sclerosis, CVA: persons with stroke

The samples differed in their extracted individual component scores. “Behavior”: CON vs. MS p = 0.010, d = 1.76; CON vs. CVA p = 0.331; MS vs. CVA p = 0.165 (Fig. 5A). “Neurological state”: CON vs. MS p = 0.046, d = 0.78; CON vs. CVA p < 0.001, d = 1.86; MS vs. CVA p = 0.017, Cohen’s d = 0.63 (Fig. 5B). “Physical state”: CON vs. MS p < 0.001, d = 1.40; CON vs. CVA p < 0.01, d = 1.76; MS vs. CVA p = 0.339 (Fig. 5C). Anovas indicated lateralized impairment (p < 0.001, ges = 0.49), hand (p = 0.043, ges = 0.56), and sex (p = 0.033, ges = 0.062) effects on “neurological state” and group (p = 0.002, ges = 0.16), lateralized impairment (p = 0.017, ges = 0.08), and hand (p = 0.032, ges = 0.06) effects on “physical state” as well as group-lateralized impairment (p = 0.025, ges = 0.07) and group-sex (p = 0.025, ges = 0.07) interactions. There were no significant factors for “behavior”.

### Differences of sensory capacity between the samples

**Fig. 6:**
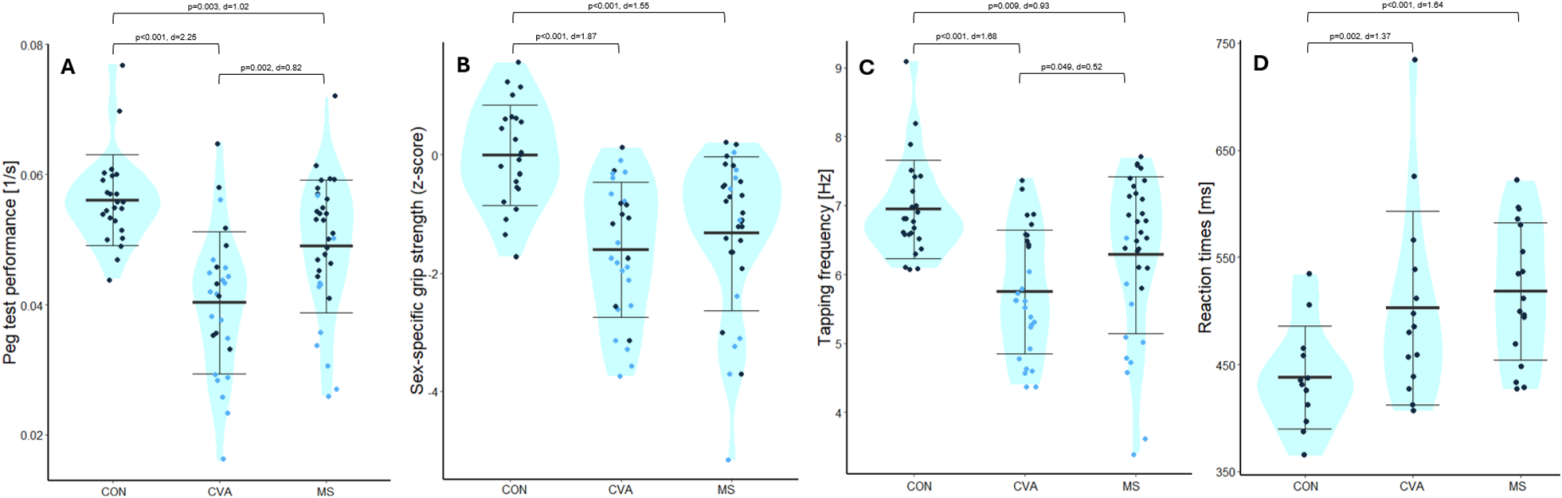
Performance distribution within the samples. Blue dot fillings indicate lateralized impairments. Bold bars indicate the mean, error bars 1 standard deviation. Comparisons on group levels are indicated with p-values and effects sizes (Glass’ delta for comparisons with CON, Cohen’s d for comparisons between MS and CVA). **A** Peg test performance as inversed trial durations. Higher values indicate better performance. **B** Grip strength as z-scores. Higher values indicate better performance. **C** Tapping frequency. Higher values indicate better performance. **D** Reaction times. Higher values indicate worse performance. CON: healthy controls, MS: persons with multiple sclerosis, CVA: persons with stroke

Significant effects of the factor group were observed in all four sensorimotor capacity tests. The anova for the peg test performance revealed significant effects of group (p = 0.025, ges = 0.10) and lateralized impairment (p < 0.001, ges = 0.25). The grip strength had effects of group (p = 0.009, ges = 0.12) and lateralized impairment (p = 0.001, ges = 0.14). The tapping frequency was impacted by lateralized impairment (p < 0.001, ges = 0.49) and hand dominance (p = 0.013, ges = 0.08). Finally, reaction times were affected by group (p < 0.001, ges = 0.18) and biological sex (p < 0.001, ges = 0.30). Post-hoc tests on sample level showed a CON vs. MS difference for the peg test performance (p = 0.003, d = 1.02), grip strength (p < 0.001, d = 1.55), tapping frequency (p = 0.009, d = 0.93), and reaction times (p < 0.001, d = 1.64). The healthy adults showed better performance in all tests. The post-hoc comparison between CON and CVA was significant for the peg test performance (p < 0.001, d = 2.25), the grip strength (p < 0.001, d = 1.87), the tapping frequency (p < 0.001, d = 1.68), and the reaction times (p = 0.002, d = 1.37). The healthy adults showed better performance in all tests. Note that individuals in the CON group were significantly younger than participants of the CVA group (Tab. 1). Post-hoc comparisons between MS and CVA was significant for the peg test performance (p = 0.002, Cohen’s d = 0.82; MS better than CVA) and the tapping frequency (p = 0.049, Cohen’s d = 0.52; MS better than CVA), but not for the grip strength (p = 0.382) or reaction times (p = 0.536). Figure 6 shows the distributions of performance within the samples, with lateralized impairments highlighted as blue dot fillings.

### Different reaction of neurological samples to neurorehabilitation

**Fig. 7:**
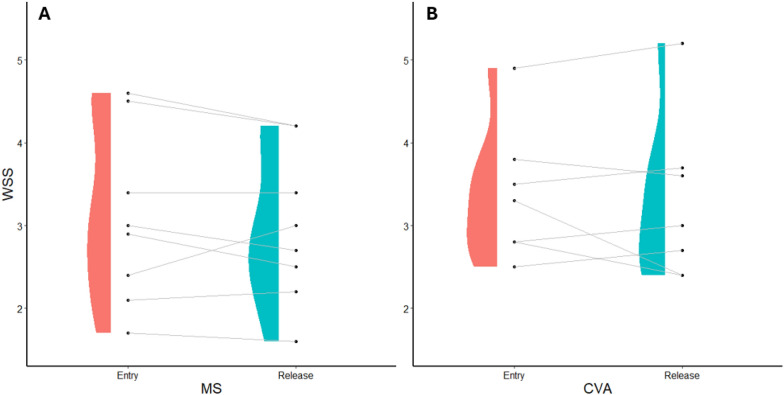
Individual changes in the WSS over the course of neurorehabilitation. The time between assessments was 10-27d. Higher values indicate a worse sensorimotor state. **A** Changes in persons with MS. **B** Changes in persons with stroke

There were no significant changes in the WSS over the course of rehabilitation (MS p = 0.430, CVA p = 0.626) (Fig. 7). However, significant improvements were observed in the peg test performance (inversed trial duration: MS p = 0.015, Cohen’s d = 0.50, −1.9s; CVA p = 0.121) and grip strength (MS p = 0.006, Cohen’s d = 0.35, + 2.1kg; CVA p < 0.001, Cohen’s d = 0.29, + 2.3kg). There were mixed results concerning reaction times (improvement: MS p = 0.003, Cohen’s d = 0.66, −41ms; worsening: CVA p = 0.005, Cohen’s d = 0.41, + 36ms). There were no effects considering motor speed (tapping frequency: MS p = 0.079; CVA p = 0.322). None of the smartwatch derived behavioral parameters revealed any changes between the two assessment days. There was a moderate, positive relationship between the time between assessments and changes in the WSS (r = 0.37, p < 0.001), indicating increments in the WSS (i.e., worse state) with longer time spent in neurorehabilitation. The time spent in neurorehabilitation was independent of the initial WSS (r = − 0.05, p = 0.859).

### Translation of sensorimotor capacity changes to behavior kinematics

**Fig. 8:**
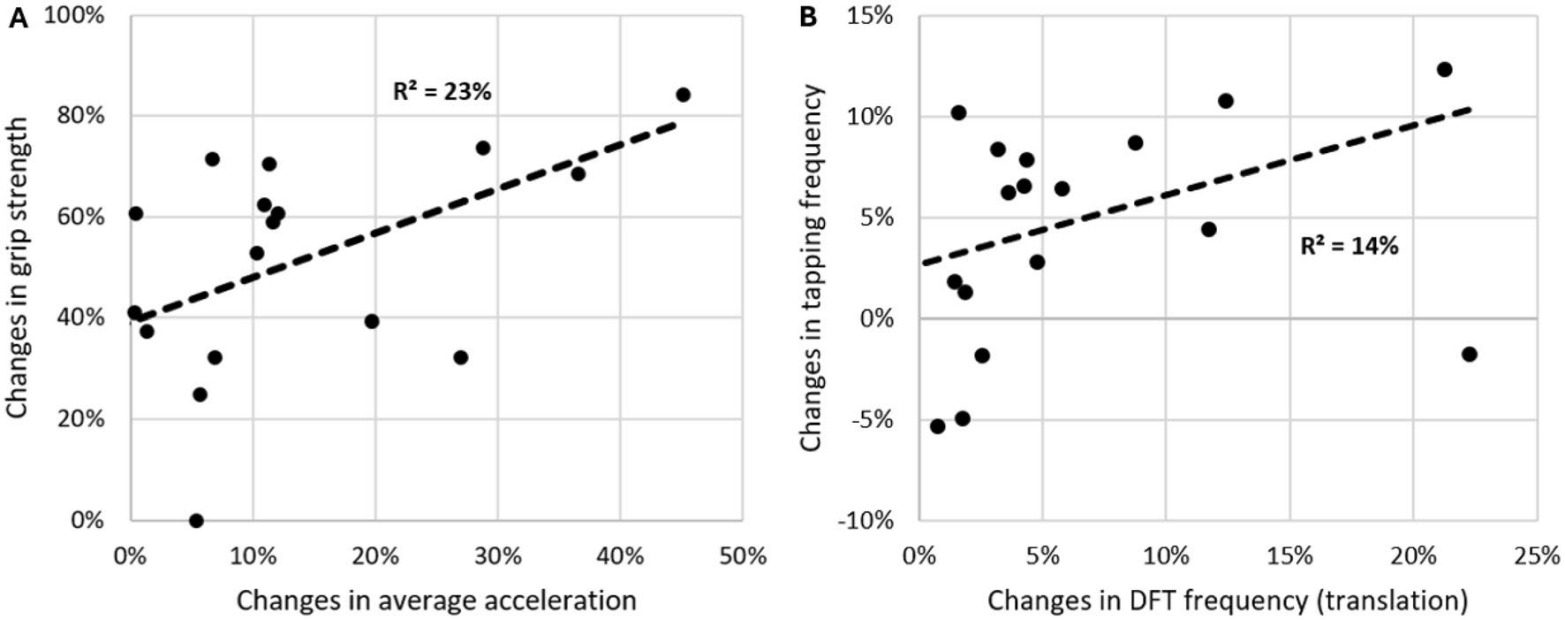
(Repeated measures) Correlations of changes in sensorimotor capacity and behavior kinematics over the course of neurorehabilitation. **A** Changes in grip strength and average acceleration. Higher values indicate improvements. **B** Changes in tapping frequency and DFT frequency of translation. Higher values indicate improvements

Furthermore, no significant associations were found between the changes in the peg test performance and the kinematics of everyday behavior. However, changes in grip strength showed a significant association with the average acceleration during activities (r = 0.48, p = 0.010) (Fig. 8A), Additionally, changes in tapping frequency were significantly correlated with DFT frequencies of translation (r = 0.38, p = 0.048) (Fig. 8B).

## Discussion

In this study, data on sensorimotor capacity and everyday behavior kinematics from healthy adults, persons with MS, and persons with stroke were collected. Persons with neurological diseases revealed less upper limb activities compared to healthy adults, yet their activities tended to be of longer duration. Moreover, persons with stroke exhibited less intense movements and persons with MS showed less coordinated movements in comparison to healthy adults. Sensorimotor capacity was weakly to moderately associated with behavior kinematics, and changes in both sensorimotor capacity and kinematics were only in two cases correlated throughout the course of neurorehabilitation. However, this association was limited to grip strength and motor speed. A PCA indicated that persons with neurological diseases show greater variability in both their everyday “behavior” and their “neurological state” and “physical state” compared to healthy adults. Notably, the factor impaired side did not load on the “behavioral” component and had only a cross-loading on the “physical state” component.

### Upper limb use

In general, behavior kinematics proved to be independent from the physical activity level measured by the step frequency. Further, there was a negative, non-linear association between the number and duration of activities, while the volume of activity was preserved (activity count multiplied by duration). To what extent this is due to the thresholds of the algorithm cannot be answered at this point. Still, a similar non-linearity in the association between capacity and use was recently reported by Hirayama et al. [32]. Lower numbers and longer durations of activities were observed in the neurological samples. In this regard, persons with impaired sensorimotor capacity appeared to interact less with themselves and their environment. Moreover, when they did engage, the interactions tended to take longer, which is in accordance with findings from analyses of ADL across various samples, including stroke, Alzheimer's disease, and advanced age [19, 20, 23]. The total time spent on activities, based on the quotient of summed activity durations and the wear time, remained consistent, accounting for approximately 10% of the total time (11.4% ± 9.9%, ranging from 1.0% to 45.1%). The total time spent on activities must be taken with care, since we observed persons during an inpatient stay and not at their homes. Therefore, the activity times could have been overestimated due to movement therapies. The observed non-linear relationship between the frequency of use and duration further supports the “threshold hypothesis” [57], which suggests that a minimum functional state of a limb is required for its integration into everyday life. In our complete sample, the peg test performance showed a weaker association with the number of activities compared to the box and block test performance reported by Schwer de Lucena et al. [57]. This discrepancy might be attributed to the relatively better performance of the neurological samples in the peg test (average 24.0s, maximum 64.4s), or to the fact that the box and block test relies more on larger translational movements required to move the hand to the designated location for interaction, although the connections between peg test performance and translational behavior kinematics in our study were moderate. When being active, persons with neurological disease showed reduced intensities of translation and lower translation frequencies (DFT), indicating slower movements. In persons with MS, we were further able to observe high maximum accelerations and angular velocities, coupled with lower frequency weights (in translation only a statistical trend). This could indicate reduced movement control, characterized by lower smoothness and impaired movement damping [35, 52], which would be supported by the reduced “behavior” component scores. Overall, more intense behavior was associated with better manual dexterity and motor speed (Table 3). As a note, in one case, the maximum acceleration, we observed no differences between the neurological samples and CON, but between MS and CVA, with MS showing significantly higher maximum accelerations than CVA. As discussed above, this could be based on reduced movement control, especially considering movement damping.

### Sensorimotor capacity

As expected, persons with neurological impairments, despite showing rather mild to moderate neurological severity (WSS), performed worse in the sensorimotor assessments. Nevertheless, it is important to acknowledge that the peg test performance, grip strength, and reaction times were not only affected by the disease but also by the “physical state” (e.g., by detraining through less use as a possible pathway). This is reflected by the effects of group (primarily related to “physical state”) and lateralized impairment (primarily related to “neurological state” but also to “physical state”); as well as the loadings on the components “neurological state” and “physical state”. “Physical state” and “neurological state” are orthogonal to each other, meaning that one observes independent dimensions. However, there were cross-loadings of the peg test and the WSS, since their scores can be affected by both dimensions. Tapping was found to be least affected by the “physical state” (Table 4: smallest loading on “physical state” and strongest loading on “neurological state”; further, an anova only showed an effect of lateralized impairment and handedness but not of group) and further revealed the strongest associations with behavior kinematics (of intensity). In prior studies, it has been shown that tapping is not only a very reliable [26] but also valid [9, 28] measure of neurological impairment.

### Effects of neurorehabilitation

Although we did not observe a significant main effect of rehabilitation on the sensorimotor state in our sample, as measured by the WSS (Fig. 7), improvements were noted in specific measures. The peg test performance and reaction times improved in persons with MS, grip strength increased in both neurological samples, while reaction times worsened in persons with stroke. It has to be kept in mind that, due to the small samples and the short time window between measurements, only moderate to strong effects can be observed. I.e., not observing significant effects in our samples should not imply that neurorehabilitation measures are not showing effects at all. When considering the components of the PCA, it was primarily the measures loading on the “physical state” that benefited from neurorehabilitation, aligning with previous findings [27]. It is important to note that significant WSS improvements were demonstrated in larger samples of persons with MS undergoing neurorehabilitation in prior studies [24, 27]. On an individual level, i.e., by means of correlational analyses, we were able to significantly associate changes in grip strength with increased intensities of translational movements (Fig. 8A) and changes in tapping frequency with quicker translational movements (Fig. 8B). For grip strength, this is in line with the correlation of mean acceleration and grip strength. Apparently, the gains in strength (e.g., by resistance training) enabled more intense movements of the arm. Improvements in motor speed, on the other hand, were translated into quicker movements. This is in line with prior findings on changes of movement kinematics in persons with stroke that improvements can be seen in improved movement smoothness, shorter movement durations, and increased average but reduced maximum intensities [52]. If future studies validate our findings, the key question should shift from whether improvements in sensorimotor capacity impact everyday behavior to how best to induce these positive changes in persons with neurological disease. At least in persons with MS, the very little to missing improvements in the “neurological state” [26] could be attributed to the generally low repetition rates and intensities that can generally be seen in neurorehabilitation [38]. Despite this, high repetition rates and intensities have shown effectiveness in MS [28], yet such approaches have not demonstrated similar effects on everyday behavior in persons with stroke [60]. Therefore, compensation, i.e., changes in the domain of the “physical state” could be a significant track to improved functioning for persons with stroke. This means that, if neurorehabilitation is not evoking changes in the “neurological state” of persons with stroke, improving the “physical state”, e.g., by increasing muscle mass, can help to nevertheless meaningfully regain function.

### Principal components

Three components were extracted by the PCA. The first component, “behavior”, describes the kinematics of activities. The factor loadings for this component indicate that high component scores are associated with short, translationally and rotationally smooth, less jerky (well-dampened, respectively) movements, as well as quick rotations of the wrist. This is very much in line with the findings of Rohrer et al. [52], reporting improved movement smoothness and average intensities, as well as shorter movement durations and reduced maximum intensities. Persons with MS and stroke exhibited considerable variability in their component scores on the “behavior” component. However, there was no significant effect of lateralized impairment, nor did sensorimotor capacity measures show any notable cross-loadings on this component. This suggests that persons tend to engage in activities that they can easily perform, avoiding those that exceed their coordinative capabilities or “comfort zone”, respectively (otherwise, one could expect an impact of lateralized impairment on the “behavior” component). Some remaining unavoidable tasks (e.g., feeding or washing hands) could then lead to the great variability, especially when the effects of the disease are not predominantly lateralized (i.e., in MS). Note that 2MPAC is assessing activities and not physical activity levels. When considering everyday behavior as a training stimulus, this variability presents a challenge.

The second component, “neurological state”, describes the neurological impairments that are indicated by the factor loadings of lateralized impairment and tapping frequency, as well as the cross-loadings of WSS, peg test, and even (slightly) age. The cross-loadings of WSS and peg test are shared with the “physical state” component and can be explained by their dependency on neurological damage and training (or “bodily fitness”). Interestingly, we also observe positive cross-loadings of behavior kinematics, specifically maximum acceleration and rotation. This could indicate that these maxima may rely on the system’s capacity to generate high forces or speeds, as evident by the loading of motor speed. Simultaneously, these high values could reflect the presence of jerky movements. Hence, it is important to interpret these metrics cautiously, considering the potential interaction of such factors. The effect of lateralized impairment is well reflected in Fig. 5B. Additionally, the effect of hand dominance was previously observed in persons with stroke [30]: It makes a difference if your dominant or non-dominant upper limb is more affected. Interestingly, as illustrated in Fig. 5B, the observed ipsilesional impaired performance (as for instance in ADL according to [19]), is not apparent in this stroke sample. This further emphasizes, with small power, the necessity of differentiating between the “neurological state” and the “physical state”. However, consider the limitations of the used clinical tests and the small sample in this study.

The third component, “physical state”, most likely describes the non-neurological impairment that can derive from detraining by non- or less use (as a potential pathway), or the bias of developing neurological diseases when having a low bodily fitness [8, 13]. Age and grip strength show the strongest factor loadings and—as discussed before—there are strong cross-loadings of the peg test performance and WSS. A cross-loading of lateralized impairment may reflect the impact of detraining by using the (more) affected upper limb less. The effect of group on the component scores is approximately twice as large as the effect of lateralized impairment, highlighting that not only detraining by non- or less use is problematic in neurological disease, but especially sedentarism, which affects both sides of the body [15, 44]. The group-lateralized impairment interaction is well reflected in Fig. 5C and it appears that detraining poses a significant greater challenge for persons with stroke compared to those with MS; the impact of lateralized impairment has different magnitudes of impact on group level, as in MS, the “physical state” of both limbs was rather comparable, while the “neurological state” strongly differed. In contrast, in CVA, both “physical state” and “neurological state” show negative effects of the factor lateralized impairment. This could be due to the commonly advanced age of persons with stroke, the sudden onset of the disease, and their reportedly high rates of depression and fatigue [46, 56], although persons with MS also show a high prevalence of affective disorders [51]. The negative impact of neurorehabilitation on reaction times of persons with stroke, compared to the positive effects observed in persons with MS, may indicate a significantly reduced physical and psychological resilience in our stroke sample. This reduced resilience could lead to greater exertion during the neurorehabilitation program, aligning with the interaction between group and lateralized impairment.

To summarize the findings from the PCA, persons with neurological disease do not only show an impaired “neurological state” but also a reduced “physical state”. Particularly in persons with MS, the impact of detraining by non- or less use or sedentarism might even be stronger than the underlying neurological impairment. This aligns with prior findings indicating that an unhealthy lifestyle serves as a predictor of greater neurorehabilitation success, which in turn is driven by implementing a healthy lifestyle during the inpatient stay [27]. Given that persons tend to mostly engage in everyday activities that are below their capacity, this vicious circle could potentially be broken by lifestyle education, intense, voluminous, and meaningful therapy sessions, and activity affordances in the home environment and community [27, 28, 33, 38, 49].

### Limitations

In this study, several limitations must be considered. First, we did not include the type of stroke (ischemic or hemorrhagic) or MS (relapsing remitting or progressive) into our analyses, neither did we consider the differences in time since stroke. This was due to our relatively small sample sizes and the number of, in our opinion, more meaningful factors like hand dominance or biological sex. However, it has been shown that the type of MS does not necessarily affect the rehabilitation success of the upper limb [34], same as time post-stroke within the range of our sample [42]. Further, Perna and Temple [48] report no meaningful effect of stroke type on the effects of neurorehabilitation. As we recorded the behavior in an inpatient setting, the behavior does not necessarily present the one at home. Due to movement therapy and a schedule, i.e., a structured day, the activity level of participants could be overestimated. Finally, it is important to note that we only recorded approximately 8h per upper limb due to the limited battery life of the smartwatches. Still, this led to the analysis of several hundreds to thousands of activities per participant. One technical aspect must be taken into account. The algorithm used a fixed interval to define the end of an activity, which may have resulted in incorrectly logging multiple activities instead of a single activity with prolonged hesitations within the execution. This could have been moderating the observed association between activity frequency (activities per minute) and activity duration.

## Conclusion

Persons with neurological disease engaged in fewer activities, but these activities tended to be of longer duration. Moreover, they not only contend with neurological impairments but also experience a decline in physical condition. In our mixed sample, neurorehabilitation predominantly enhanced the “physical state” rather than the “neurological state”. These improvements were partially translated into (inpatient) everyday life kinematics. Overall, sensorimotor capacity and behavior were not strongly associated in our samples comprising individuals with no and mild to moderate disease severity. In (inpatient) everyday life, persons appeared to stay within their sensorimotor “comfort zone”. Therefore, improving the sensorimotor capacity could indeed increase the probability of engaging in more demanding activities and prevent persons from becoming dependent in everyday life. Vice versa, everyday life is not an effective measure to increase sensorimotor capacity. Our analyses emphasize that kinematic parameters of activity durations, intensities, and smoothness can provide valuable insights into the (inpatient) everyday life behavior of persons with neurological disease. Further, we were able to introduce an algorithm for the detection of activities that apparently allowed to analyze behavior independent of the physical activity volume.

## Data Availability

Data is available on reasonable request from the corresponding author.
